# Proteomics and Its Combined Analysis with Transcriptomics: Liver Fat-Lowering Effect of Taurine in High-Fat Fed Grouper (*Epinephelus coioides*)

**DOI:** 10.3390/ani14142039

**Published:** 2024-07-11

**Authors:** Yu Zhou, Fakai Bai, Ruyi Xiao, Mingfan Chen, Yunzhang Sun, Jidan Ye

**Affiliations:** Xiamen Key Laboratory for Feed Quality Testing and Safety Evaluation, Fisheries College of Jimei University, Xiamen 361021, China; 202212951062@jmu.edu.cn (Y.Z.); 201911908010@jmu.edu.cn (F.B.); 201911908023@jmu.edu.cn (R.X.); 202111908029@jmu.edu.cn (M.C.); jmusunyunzhang@163.com (Y.S.)

**Keywords:** taurine, liver fat metabolism, proteomics, omics association analysis, *Epinephelus coioides*

## Abstract

**Simple Summary:**

Taurine has a wide range of biological functions in vertebrates but does not participate in protein synthesis. Recent studies showed taurine exhibits an important role in the regulation of fat metabolism in fish and has the effect of reducing liver fat accumulation in high-fed fish. However, it is still unclear how taurine exerts the effect and the underlying metabolic mechanism of taurine intervention. In this study, we conducted an experiment to investigate the fat-lowering effect of taurine on orange-spotted groupers at the proteomic level. Furthermore, we performed an integrated analysis of transcriptomics and proteomics and excavated the key genes and key proteins involved in the regulation of liver fat metabolism, in an attempt to better understand the intrinsic connection between the transcriptional and translational levels and interpret the trajectories in terms of the biological phenomena in a more comprehensive way after taurine intervention on high-fat fed orange-spotted groupers.

**Abstract:**

In order to understand the intervention effect of taurine on liver fat deposition induced by high fat intake in the orange-spotted grouper (*Epinephelus coioides*), we performed proteomic analysis and association analysis with previously obtained transcriptomic data. Three isoproteic (47% crude protein) diets were designed to contain two levels of fat and were named as the 10% fat diet (10F), 15% fat diet (15F), and 15% fat with 1% taurine (15FT). The 10F diet was used as the control diet. After 8 weeks of feeding, the 15F diet exhibited comparable weight gain, feed conversion ratio, and hepatosomatic index as the 10F diet, but the former increased liver fat content vs. the latter. Feeding with the 15FT diet resulted in an improvement in weight gain and a reduction in feed conversion ratio, hepatosomatic index, and liver fat content compared with feeding the 15F diet. When comparing liver proteomic data between the 15F and 15FT groups, a total of 133 differentially expressed proteins (DEPs) were identified, of which 51 were upregulated DEPs and 82 were downregulated DEPs. Among these DEPs, cholesterol 27-hydroxylase, phosphatidate phosphatase LPIN, phosphatidylinositol phospholipase C, and 6-phosphofructo-2-kinase were further screened out and were involved in primary bile acid biosynthesis, glycerolipid metabolism, the phosphatidylinositol signaling system, and the AMPK signaling pathway as key DEPs in terms of alleviating liver fat deposition of taurine in high-fat fed fish. With the association analysis of transcriptomic and proteomic data through KEGG, three differentially expressed genes (*atp1a*, *arf1_2*, and *plcd*) and four DEPs (CYP27α1, LPIN, PLCD, and PTK2B) were co-enriched into five pathways related to fat metabolism including primary bile acid synthesis, bile secretion, glycerolipid metabolism, phospholipid D signaling, or/and phosphatidylinositol signaling. The results showed that dietary taurine intervention could trigger activation of bile acid biosynthesis and inhibition of triglyceride biosynthesis, thereby mediating the liver fat-lowering effects in high-fat fed orange-spotted grouper. The present study contributes some novel insight into the liver fat-lowering effects of dietary taurine in high-fat fed groupers.

## 1. Introduction

As is well known, fat is an essential nutrient for normal growth and development of fish due to its cell structural framework and the fact that it provides fuel for cellular metabolism [1]. Moreover, fat is highly used by fish [2], and the energy utilization efficiency of the digested fat is approximately 80% (64% for digested protein and 58% for digested carbohydrates, respectively) [3]. Fat also has a protein-sparing effect in fish [4,5,6]. As a result, this drives fish farmers to pursue excessive use of fat as an effective energy source in aquafeeds to reduce the consumption of high-quality proteins for fuel, promoting feed utilization [7]. However, the feeding practice of fish has shown that prolonged intake of high-fat feed could cause visceral fat accumulation and fatty liver of fish [8] and, in severe cases, metabolic disorders including fatty liver syndrome [9,10,11]. Therefore, high-fat feed-induced fatty liver, the chronic hepatic disease relevant to nutritional metabolic syndrome, has become one of the important issues in current intensive aquaculture. In order to find an effective prevention and control method to address this threat, there is a need to have a deep understanding of the high-fat feed induced fatty liver in fish and its mechanism.

Taurine, a conditionally indispensable amino acid of farmed fish, displays a valuable potential application in solving this problem [12,13]. It is now clear that taurine has the potential to control fatty liver by relieving fat metabolism disorders of fish with dietary taurine intervention [14,15]. The fat-lowering effect of taurine has been confirmed with effective taurine intervention on fish fed high-fat diets in recent years [12,13,16,17,18,19]. However, it is still unclear how taurine regulates the fat-lowering effect and what role it plays in the fat metabolism of fish. On this issue, our research team conducted a series of studies to investigate the intervention effects of taurine in vitro and in vivo and its regulatory mechanism in high-fat fed orange-spotted grouper (*Epinephelus coioides*) via transcriptomics, lipidomics, and metabolomics in recent years [20,21,22,23]. Data from our recent lipidomic analysis show that the reduction in liver fat accumulation via dietary taurine addition may be realized through decreasing the contents of TGs containing 18:2n-6 at the *sn-2* and *sn-3* positions and through promoting the anti-inflammatory capacity of groupers [20]. In another study, the taurine-conjugated BAs have a higher ability to accelerate fat emulsification and absorption than glycine-conjugated and other BAs in the fish species [23]. Moreover, our recent studies in vivo and in vitro using transcriptomics showed that the reduction in liver fat accumulation was attributed to the fact that dietary taurine addition enhanced the synthesis of endogenous taurine in the liver, accelerated BA transport and insulin secretion, thus promoting fatty acid β−oxidation efficiency [21,22].

In the present study, we conducted an experiment regarding taurine intervention in high-fat fed orange-spotted grouper. With these results, we further investigated the fat-lowering effect of taurine on the fish species and elucidated its regulatory mechanism in fat metabolism at the proteomic level. In addition, we performed an integrated analysis of transcriptomics and proteomics based upon our existing transcriptomic and proteomic data and excavated the key genes and key proteins involved in the regulation of liver fat metabolism, in an attempt to better understand the intrinsic connection between the transcriptional and translational levels and interpret the trajectories in terms of the biological phenomena in a more comprehensive way after taurine intervention on high-fat fed orange-spotted grouper [24,25]. This study provides some new insights into the prevention and treatment of nutritional metabolic diseases (fatty liver syndrome) in fish.

## 2. Materials and Methods

### 2.1. Experimental Diets

The optimal levels of taurine and fat in feed were 1.0% and 10%, respectively, for the normal growth of orange-spotted grouper in our previous research and those of others [26,27]. Thus, in this experiment, three isoproteic (47% crude protein) semi-purified diets were prepared using casein and gelatin as the main protein ingredients and fish and soy oils and soy lecithin as the major fat ingredients, as previously described in our recent research [22], that is, control diet (10% fat diet), high-fat diet (15% fat diet), and high-fat diet with 1% taurine (15% fat diet with 1% taurine), designated 10F, 15F, and 15FT, respectively (Table 1). The mixed powder feeds were made into sinking pellets, dried, and then stored at −20 °C until use, according to our previous practice [12].

### 2.2. Growth Trial

The grouper juveniles were obtained from a local hatchery (Zhangpu county, Fujian, China). Prior to the start of the trial, the fish were maintained with a commercial diet (46.1% crude protein and 9.6% crude fat) in a three-week acclimation. A total of 270 groupers with an initial wet weight of about 10.5 g were randomly distributed into nine tanks within a recirculating aquaculture system at a water flow rate of 8 L/min per tank. The groupers of nine tanks were arranged into three treatments, each with triplicate 300 L tanks at a stock density of 30 fish per tank. The groups of triplicate tanks were hand-fed twice daily (8:30 and 18:30) across a feeding period of 8 weeks. Excess feed was collected 30 min after each meal to determine feed intake. During the feeding period, the daily rearing management followed our previous practice [11].

### 2.3. Sample Collection

At the end of the 8-week feeding trial, five fish were randomly sampled from each tank and sacrificed with an overdose of MS-222 solution (tricaine methanesulfonate, Sigma-Aldrich Shanghai Trading Co., Ltd., Shanghai, China), followed by fish count and batch weighing, and were recorded on a wet weight basis to determine percent weight gain and feed conversion ratio. After completing the weighing, liver was removed to calculate the hepatosomatic index (HSI) and pooled by tank in a tube for determining liver fat content. The same batch of five fish per tank were randomly caught and dissected to aseptically remove livers and were pooled into one tube by tank and then stored at −80 °C for the subsequent extraction of protein.

### 2.4. Protein Digestion and Peptide TMT Labeling

The volume ratio of liver tissue to lysis buffer SDT in the homogenate was one to four. SDT (4% (*w*/*v*) SDS, 100 mM Tris/HCl, pH = 7.6, 0.1M DTT) buffer was used for liver sample lysis and protein extraction. The supernatant (protein solution) was collected. Briefly, 200 μg of protein solution per sample was added to a dithiothreitol solution (DTT, Sigma-Aldrich Shanghai Trading Co., Ltd., Shanghai, China) to make a mixed solution with a final concentration of 5 mmol/L, and the reaction was maintained at 56 °C for 30 min, followed by the addition of iodoacetamide (IAA, Sigma-Aldrich Shanghai Trading Co., Ltd., Shanghai, China), to make a mixed solution with a final concentration of 11 mmol/L, and was then kept at room temperature for 30 min in the dark. The reaction was terminated with triethylamine-carbonate buffer solution (TECS). The amount of protein in the liver sample was quantified with the BCA Protein Assay Kit (Bio-Rad., New York, NY, USA). Protein digestion by trypsin was performed according to the filter-aided sample preparation (FASP) procedure described by Matthias Mann [28]. The digest peptides of each liver sample were desalted on C18 Cartridges (Empore™ SPE Cartridges C18 (standard density), bed ID7 mm, volume 3 mL, Sigma-Aldrich Shanghai Trading Co., Ltd., Shanghai, China) and then concentrated via vacuum centrifugation and reconstituted in 40 µL of 0.1% (*v*/*v*) formic acid [29]. The resulting peptide mixture of each liver sample (100 μg) was labeled using TMT (tandem mass tag) reagent according to the manufacturer’s instructions (Thermo Scientific, Xiamen, China).

### 2.5. High-PH Reversed-Phase Peptide Fractionation

Peptides that have been labeled with the TMT labeling kit, and then mixed in equal parts. The gradient elution separation of aliquots of labeled peptides for each liver sample was performed using the high-pH reversed-phase peptide fractionation kit (Thermo Scientific). The specific procedures refer to Zhang’s description [30]. Firstly, column equilibration was achieved using acetonitrile and 0.1% trifluoroacetic acid. Then, the labeled peptide mixture was added with pure water, followed by centrifugation at low speed for desalination. Finally, step-gradient elution of column-bound peptides was performed with increasing concentrations of high-pH acetonitrile solution. Each eluted peptide sample was then vacuum dried. The lyophilized peptide sample was resolved in 12 μL of 0.1% TFA, and the peptide concentration was determined at OD280.

### 2.6. LC-MS/MS Analysis

LC-MS/MS data collection refers to the method in [30]. Briefly, a HPLC liquid-phase system (ASY-nLCTM 1200 nm version) was used for the separation of each sample. Buffers A and B were 0.1% formic acid and 0.1% formic acid acetonitrile (84% acetonitrile), respectively. After the column equilibrium was achieved with 95% buffer solution A, the sample was injected via the autosampler to the loading column (Thermo Scientific Acclaim PepMap100, 100 µm × 2 cm, nanoViper C18) and separated through the analytical column (Thermo Scientific EASY column, 10 cm, ID 75 µm, 3 µm, C18-A2) with a flow rate of 300 nL/min.

After completion of chromatographic separation, the peptides were analyzed using a Q-Exactive mass spectrometer (Thermo Scientific). The detection conditions were set as follows: detection mode: positive ion; parent ion scan range: 300–1800 *m*/*z*; first order mass spectrometry resolution: 70,000 at 200 *m*/*z*; automatic gain control target setting: 100,000; maximum injection time: 50 ms; and dynamic exclusion time: 60 s.

Mass charge ratios of polypeptides and polypeptide fragments were collected according to the following methods: 20 fragment maps were collected for each full scan. The specific parameter settings were as follows: MS2 Activation Type: HCD (higher-energy collision-induced dissociation); isolation window: 2 *m*/*z*; resolution of the secondary mass spectrometry: 17,500 at *m*/*z* 200; normalized collision energy: 30 eV; and lower fill ratio: 0.1%.

### 2.7. Database Search and Data Analysis

After completion of the LC-MS/MS analysis, the data in raw files were checked in databases through the software Mascot2.2 (Matrix Science, London, UK) and the software Proteome Discoverer1.4 (Thermo Fisher Scientific, San Jose, CA, USA), and the TMT labeling quantitative analysis was performed. The database search method described by Lin was performed [31]. Briefly, the MS raw data for each sample were searched using the Mascot engine (Matrix Science, London, UK; version 2.2) embedded into Proteome Discoverer 1.4 software (Thermo Electron, San Jose, CA, USA) for identification and quantitation analysis. The Mascot search parameters and instructions are as follows: Max missed cleavages: 2; fixed modifications: Carbamidomethyl (C), iTRAQ 4/8 plex (N-term), iTRAQ 4/8 plex (K), TMT 6/10/16 plex (N-term), TMT 6/10/16 plex (K); variable modifications: oxidation (M), Ox iTRAQ 4/8 plex (Y), TMT 6/10/16 plex (Y); peptide mass tolerance: ±20 ppm; fragment mass tolerance: 0.1 Da; database pattern: decoy; and peptide FDR ≤ 0.01.

The differentially expressed proteins (DEPs) were filtered under the criteria of *p*-value < 0.05 and fold change (FC) > 1.2-fold for upregulation or less than 0.83 for downregulation [32]. The number of up- and downregulated DEPs of liver in the comparison group was finally obtained.

### 2.8. Bioinformatics Analysis

Blast2GO (https://www.blast2go.com/) was used to annotate functions of DEPs by GO, which was categorized into three main groups: biological process, molecular function, and cellular component [33]. The DEPs were numbered after GO function annotation of all DEPs [34]. The Kyoto Encyclopedia of Genes and Genomes (KEGG) pathway enrichment was performed on DEPs through the database for the KEGG pathway by using the KAAS (KEGG Automatic Annotation Server) online service tool to associate the KEGG ORTHOLOG (KO) numbers of the DEPs to the KEGG pathway [35].

### 2.9. Parallel Reaction Monitoring (PRM) Analysis

To further verify the reliability of DEPs with TMT analysis, eight randomly selected DEPs were subjected to PRM validation (Table 2). Peptide information was imported into the software Xcalibur4.3 (Thermo Fisher Scientific, San Jose, CA, USA) for PRM method setup. Approximately 1 μg of peptide taken from each sample and 20 fmol of labeled peptide (PRTC: GISNEGQNASIK) were mixed. According to the method in [36], gradient separation of peptides was performed using a high-performance liquid chromatography system with buffer solution A: 0.1% formic acid aqueous solution and buffer solution B: 0.1% formic acid acetonitrile aqueous solution (84% acetonitrile). After separation of peptides, the target peptides were analyzed via PRM mass spectrometry using a Q-Exactive HF mass spectrometer (Thermo Scientific). The specific parameters were as follows: primary MS scan range: 300–1800 *m*/*z*; MS resolution: 60,000 (*m*/*z* 200); automatic gain control target setting: 3,000,000; maximum injection time: 200 ms; analysis time: 60 min; detection mode: positive ions; 20 MS2 scans at each primary MS scan; PRM scans (MS2 scans), isolation window: 1.6 Th; MS resolution: 30,000 (*m*/*z* 200); MS2 activation type: HCD; and normalized collision energy: 27 ev. A total of 9 samples from three groups were subjected to PRM detection, and the PRM raw files were analyzed on the software Skyline 3.5.0 (MacCoss Lab, University of Washington, Seattle, WA, USA).

### 2.10. Association Analysis of Transcriptomic and Proteomic Data

Based on the transcriptomic data previously obtained in our laboratory and the proteomic data currently obtained, special attention needs to be paid to the DEGs and DEPs enriched through the KEGG database, as well as their related pathways. The association analysis of DEGs and DEPs was performed to identify any common pathways between DEGs and DEPs, or to find pathways that are correlated upstream and downstream, or the pathways that have common metabolites. To this end, a network diagram was constructed to demonstrate the joint participation of DEPs and DEGs in liver fat metabolism after taurine intervention on a high-fat fed grouper.

### 2.11. Statistical Analysis

Data are presented as means ± SD with *n* = 3 for growth weight, feed conversion ratio, and liver fat content, while hepatosomatic index data are presented as means ± SD with *n* = 15. Significant differences among dietary treatments were analyzed with one-way ANOVA and Student–Neuman–Keuls multiple comparison test after data were tested for normality and homogeneity of variance with the Kolmogorov–Smirnov test and Levene’s test in SPSS Statistics 22.0 (SPSS, Michigan Avenue, Chicago, IL, USA). Student’s *t*-test was applied for comparison between the 15F diet and 15FT diet. The processing of histograms was performed using GraphPad prism 9.0 (San Diego, CA, USA) software. A *p*-value < 0.05 was considered statistically significant.

## 3. Results

### 3.1. Growth Performance and Liver Fat Contents

**Figure 1:**
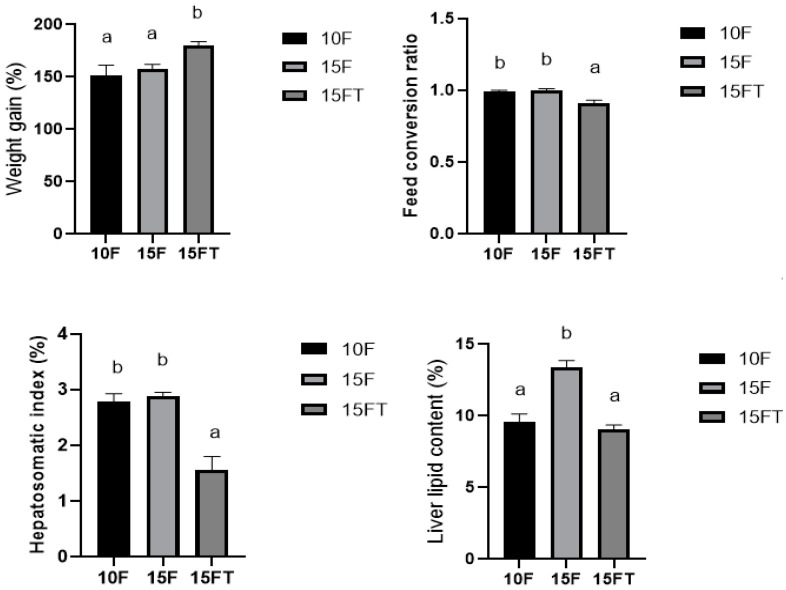
Effects of experimental diets on growth performance and liver fat contents of groupers. Groups: 10F, 10% fat diet; 15F, 15% fat diet; 15FT, 15% fat diet with 1% taurine. Weight gain (%) = 100 × (final body weight − initial body weight)/initial body weight; feed conversion ratio = feed intake/wet weight gain; hepatosomatic index (%) = 100 × (liver weight/wet body weight); values for WG, FCR, and liver fat content are presented as the means ± SD (*n* = 3 tanks); values of hepatosomatic index are presented as the means ± SD (*n* = 15 fish); values on the bar with different lowercase letter superscripts indicate significant differences (*p* < 0.05); statistical analysis was performed using one-way ANOVA, followed by Student−Neuman−Keuls multiple comparison test.

The growth performance of groupers is shown in Figure 1. There was no difference in weight gain, feed conversion ratio, and hepatosomatic index between the 10F and 15F diets (*p* > 0.05). However, the 15FT diet resulted in improved weight gain and reduced feed conversion ratio and hepatosomatic index compared with the 15F diet (*p* < 0.05).

The liver fat content in the 15F diet was significantly higher (*p* < 0.05) than that in the 10F diet. Fish fed the 15FT diet had lower (*p* < 0.05) liver fat content compared with those fed the 15F diet, and exhibited comparable liver fat content to those of fish fed the 10F diet (*p* > 0.05).

### 3.2. Proteome Profiling

Compared with the 10F diet, a significantly higher liver content was observed in the 15F diet fed fish, and the distinct intervention effect of taurine on the 15FT diet fed fish was also observed. Therefore, we chose 15F and 15FT as the comparison group to analyze the subsequent fat metabolic response after taurine intervention at the proteomic level.

**Figure 2:**
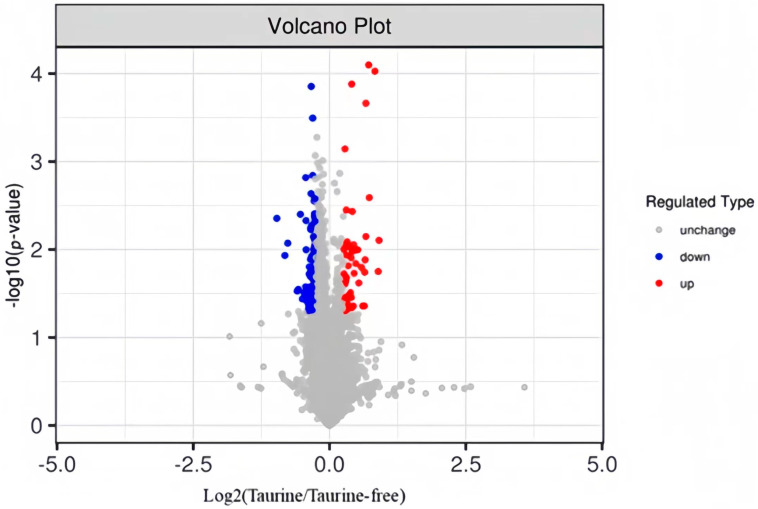
Volcano plot for identified and quantified proteins in the present study. Red and blue dots indicate proteins that were differentially expressed, up− or downregulation, respectively, in the 15% fat diet with 1% taurine group versus the 15% fat diet group.

A total of 133 DEPs were filtered by comparing the 15FT and 15F groups, of which 51 proteins were significantly upregulated and 82 proteins were significantly downregulated in the taurine group compared with the non-taurine group (Figure 2).

### 3.3. Bioinformatics Analysis

**Figure 3:**
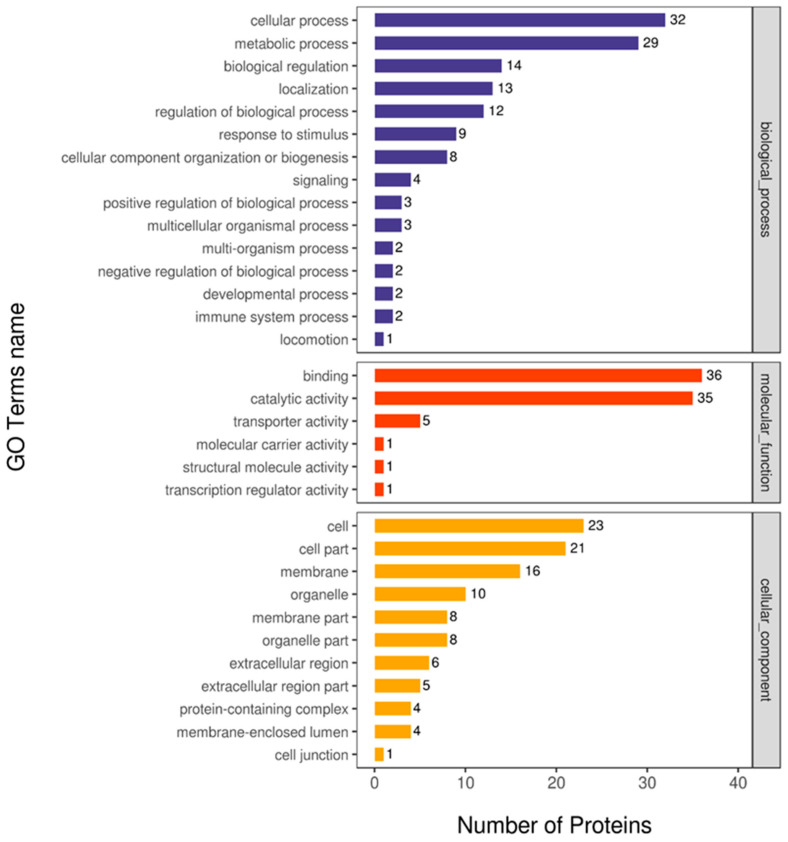
GO annotation statistics of differentially expressed proteins in 15% fat diet with 1% taurine group versus 15% fat diet group.

GO analysis included biological process, molecular function, and cellular component. As shown in Figure 3, the dominant subcategories were cellular process and metabolic process in the category of biological processes, the binding activity and catalytic activity appeared dominant in the category of molecular function, and the cell and cell part showed the highest percentage in cellular component.

**Figure 4:**
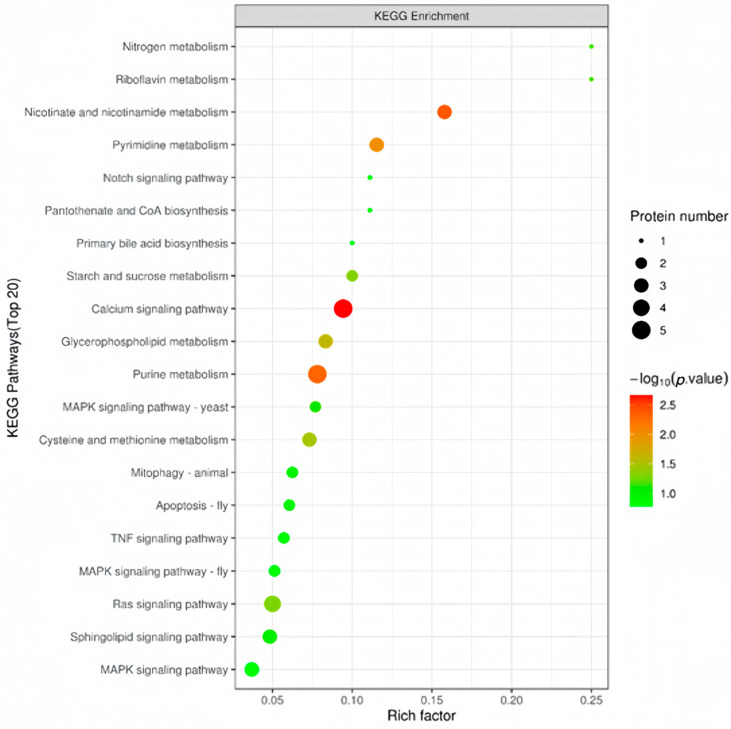
Bubble plot of KEGG pathway enrichment in 15% fat diet with 1% taurine group versus 15% fat diet group.

The DEPs were subjected to a KEGG pathway enrichment analysis. Results are shown in Figure 4. In the present study, we mainly focused on metabolic pathways directly related to fat metabolism, such as primary bile acid synthesis and glycerophospholipid metabolism, although many DEPs were enriched in metabolic pathways such as calcium signaling and purine metabolism.

### 3.4. Protein Pathways Related to Fat Metabolism after Taurine Intervention

**Figure 5:**
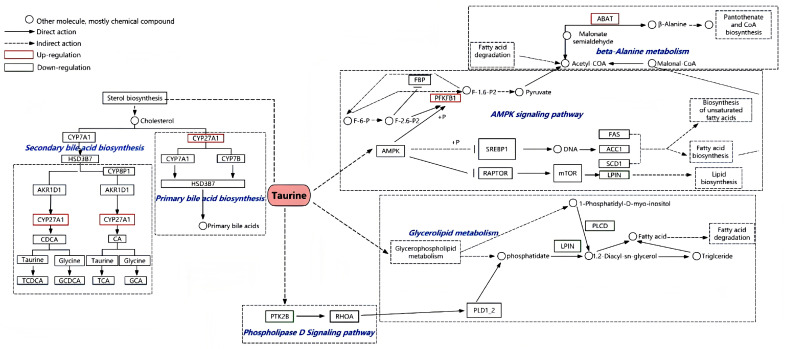
Network diagram of proteins related to fat metabolism in the liver of groupers after taurine intervention. CYP27A1, cholesterol 27-hydroxylase; PFKFB1, 6-phosphofructo-2-kinase; ABAT, 4-aminobutyrate aminotransferase; PCBD, 4a-hydroxytetrahydrobiopterin dehydratase; PLCD, phosphatidylinositol phospholipase C; LPIN, phosphatidate phosphatase LPIN; SREBP1, sterol regulatory element-binding protein 1; FAS, fatty acid synthase; ACC1, acyl-CoA carboxylase1; mTOR, mammalian target of rapamycin; PLD1_2, phospholipase D1_2; FBP, fructose-1,6-bisphosphatase I; AMPK, AMP-activated protein kinase; RAPTOR, regulatory-associated protein of mTOR; RHOA, Ras homolog gene family, member A; PTK2B, Protein Tyrosine Kinase 2 Beta; HSD3B7, hydroxy-delta-5-steroid dehydrogenase, 3 beta- and steroid delta-isomerase 7; AKR1D1, aldo-keto reductase family 1, member D1; CDCA, chenodeoxycholic acid; CA, cholic acid; TCDCA, taurochenodeoxycholic acid; GCDCA, glycochenodeoxycholic acid; TCA, taurocholic acid; GCA, glycocholic acid.

After KEGG pathway enrichment analysis of DEPs, the major protein pathways that were involved in fat metabolism included the following: glycerophospholipid metabolism, primary bile acid biosynthesis, phospholipase D signaling pathway, AMPK signaling pathway, etc. The results are shown in Table 3. Thus, the liver protein network related to fat metabolism was constructed by comparing the 15FT diet versus the 15F diet (Figure 5).

### 3.5. Validation of TMT Results with PRM

**Figure 6:**
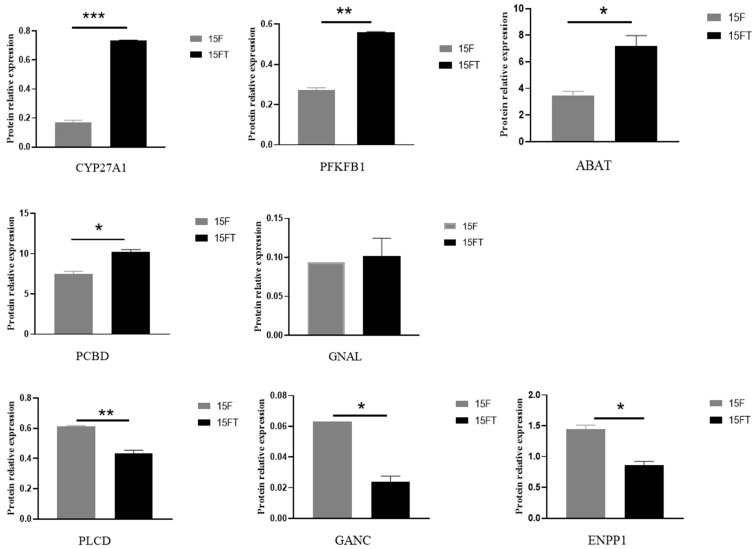
PRM verification of the expression levels of target proteins in the liver. Values are expressed as the means ± SD (n = 3). Asterisks (*, ** and ***) represent significant differences with *p* < 0.05, *p* < 0.01, and *p* < 0.001, respectively. CYP27A1, cholesterol 27-hydroxylase; PFKFB1, 6-phosphofructo-2-kinase; PLCD, Phosphatidylinositol phospholipase C; GNAL, guanine nucleotide-binding protein G subunit alpha; GANC, neutral alpha-glucosidase C; ABAT, 4-aminobutyrate aminotransferase; ENPP1, ectonucleotide pyrophosphatase; PCBD, 4a-hydroxytetrahydrobiopterin dehydratase.

Eight DEPs were subjected to PRM analysis to validate the reliability of TMT results. As is shown in Figure 6, dietary taurine addition upregulated the liver expression levels of cholesterol 27-hydroxylase (CYP27A1), 6-phosphofructo-2-kinase (PFKFB1), 4-aminobutyrate aminotransferase (ABAT), and 4a-hydroxytetrahydrobiopterin dehydratase (PCBD) but downregulated the liver expression levels of phosphatidylinositol phospholipase C (PLCD), neutral alpha-glucosidase C (GANC), and ectonucleotide pyrophosphatase (ENPP1_3); however, guanine nucleotide-binding protein G subunit alpha (GNAL) was not affected by dietary taurine addition. There were the same trends of liver expression changes for the above seven proteins subjected to PRM analysis, except GNAL, as those subjected to TMT analysis after high-fat fed fish were intervened with taurine. These results indicated that the high-throughput data through TMT analysis was of high quality and the screening results were reliable in this study.

### 3.6. Association Analysis of DEGs and DEPs

There were three DEGs (*atp1a*, *plcd*, and *arf1_2*) and four DEPs (CYP27A1, LPIN, PLCD, and PTK2B) that were associated with five KEGG pathways such as primary BA synthesis, bile secretion, glycerolipid metabolism pathway, phospholipase D signaling pathway, and the phosphatidylinositol signaling pathway (Table 4).

**Figure 7:**
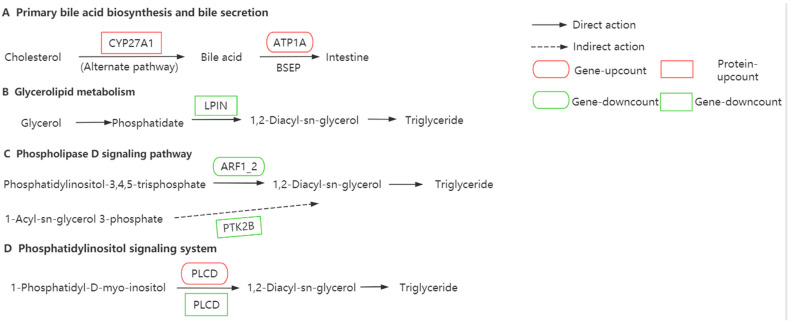
The network diagram of differentially expressed genes and differentially expressed proteins. CYP27A1, cholesterol 27-hydroxylase; ATP1A, sodium/potassium−transporting ATPase subunit alpha; LIPN, phosphatidate phosphatase LPIN; ARF1_2, ADP−ribosylation factor 1/2; PTK2B, protein tyrosine kinase 2 beta; PLCD, phosphatidylinositol phospholipase C.

As shown in Figure 7, the key protein CYP27A1 and the two key genes *atp1a* and *plcd* were upregulated by dietary taurine intervention and were co-enriched in the primary BA synthesis, bile secretion pathways, or/and phosphatidylinositol signaling pathway, promoting BA synthesis and transport. Meanwhile, the three key proteins LPIN, PLCD, and PTK2B and the key gene *arf1_2* were downregulated by dietary taurine intervention and were co-enriched in the glycerolipid metabolism pathway, phospholipase D signaling pathway, or/and phosphatidylinositol signaling pathway, resulting in a reduction in TG synthesis. The results showed that dietary taurine addition in 15% high-fat diets could improve liver fat metabolism of groupers through accelerating BA synthesis and transport and inhibiting TG synthesis.

## 4. Discussion

The results of our present study showed that the growth rate and feed utilization of groupers did not differ as the dietary fat level was increased from 10% to 15%, which indicates that groupers have a high tolerance to dietary fat. Similar results were observed in previous studies with aquatic animals [37] such as black sea bream [38] and large yellow croaker [39]. However, feeding a 15% fat diet led to increased liver fat deposit compared with feeding a 10% fat diet in this study. The increase in liver fat deposit was also observed in high-fat fed fish in many previous studies [8,39,40,41,42,43,44]. Interestingly, our current study showed a significant effect of reducing liver fat, accompanied with improved growth rate and feed utilization after intervention with taurine on 15% fat fed groupers, as evidenced by previous studies with other fish species such as *Monopterus albus*, California yellowtail, and yellowfin seabream [13,18,45]. Concomitantly, the reduced hepatosomatic index was also observed in the present study and other previous studies after intervention with dietary taurine addition in high-fat fed fish such as California yellowtail, yellowfin seabream, white grouper, turbot, and rice field eel [18,45,46,47,48].

Given the aforementioned effects of dietary taurine on reducing liver fat in fish, there may be a major regulation of taurine in liver fat metabolism at the proteomic level. For this purpose, we conducted proteomic analysis for the first time on the liver fat-lowering effect of dietary taurine in high-fat fed groupers and the possible molecular mechanisms involving fat metabolism. The results showed that a total of 133 DEPs were identified in the comparison between the 15% fat diet group and the 15% fat + 1% taurine group, of which 51 were upregulated and 82 proteins were downregulated. The KEGG pathway analysis on these DEPs showed that the DEPs were mainly enriched in primary bile acid synthesis, glycerophospholipid metabolism, the phosphatidylinositol signaling system, and the AMPK signaling pathway, which was found to be directly related to liver fat metabolism [49,50], and four DEPs (cholesterol 27-hydroxylase, phosphatidate phosphatase LPIN, 6-phosphofructo-2-kinase, and phosphatidylinositol phospholipase C) directly participated in the liver fat metabolism process after dietary taurine intervention in high-fat fed groupers. The findings of the DEPs and signaling pathways will provide a better understanding of the molecular regulation mechanisms of taurine in fat metabolism in fish.

It is clear that bile acids (BAs) are major components of bile and play a vital role in fat metabolism in mammals and fish [39,51]. They can promote fat transport and absorption in the intestine [52]. Taurine-conjugated BAs are the major form of BAs in finfish [14,23,53,54]. The reduction in BAs caused by dietary taurine addition indicates a reduction in their re-absorption in the intestine of finfish [23]. On the other hand, BAs also act as ligands to activate farnesoid X receptors (FXRs) [55,56]. The activated FXRs can reduce lipogenesis and promote fatty acid β-oxidation through inhibiting the transcription of sterol regulatory element-binding protein 1c and carbohydrate response element-binding protein [57]. This is crucial for the regulation of glucose and fat homeostasis and cellular inflammatory pathways [57]. The synthesis of BAs is regulated by the rate-limiting enzymes CYP7A1 and CYP27A1 [51,56], and the resultant taurine-conjugated BAs converted from cholesterol are highly hydrophilic, which enhances the solubility and excretion of cholesterol [58]. CYP27A1 belongs to a family of cytochrome P450 enzymes that converts cholesterol into 25(R)-26- hydroxycholesterol in the alternative pathway of BA synthesis [55,56]. 25(R)-26- hydroxycholesterol can be 7α-hydroxylated to 3β, 7α-dihydroxy-5-cholestanoic acid by oxysterol 7alpha-hydroxylase for synthesis of chenodeoxycholic acid (CDCA) and cholic acid (CA) [56]. CDCA and CA can undergo conjugation with glycine or taurine prior to secretion in the bile to form glycocholic acid, glycochenodeoxycholic acid, taurocholic acid (TCA), and taurochenodeoxycholic acid (TCDCA) [56]. In the present study, CYP27A1 expression was upregulated by dietary taurine addition and was enriched in the primary BA synthesis pathway (Figure 5), which stimulated cholesterol conversion to conjugated BAs in the liver, reflecting the hypocholesterolemic effect of taurine [54], thereby exhibiting a fat-reducing effect.

In the present study, the expression of both phosphatidate phosphatase LPIN (LPIN) protein and phosphatidylinositol phospholipase C (PLCD) protein was downregulated in the 15% fat diet with 1% taurine vs. the 15% fat diet, and they were enriched in glycerolipid metabolism and the phosphatidylinositol signaling system (Figure 5), respectively. In the synthesis of neutral TG in animals, glycerol 3-phosphate is converted into phosphatidic acid through transacylation, which then undergoes dephosphorylation by LPIN to form diacylglycerol (DAG), in turn being converted into TG through transacylation [49]. The LPIN family acts on the third step in the pathway to form DAG by dephosphorylating phosphatidic acid [59]. Lack of LPIN1 expression could affect total cholesterol accumulation in mouse adipose tissue [60,61]. Moreover, PLCD can hydrolyze the phosphatidylinositol 4,5-bisphosphate (PI (4,5) P2) into DAG and inositol 1,4,5-trisphosphate [62]. The downregulated PLCD prohibits the conversion of PI (4,5) P2 to DAG. Therefore, the reduction in TG synthesis in the liver is attributed to the downregulation of expression of LPIN and PLCD in the glycerolipid metabolism pathway and phosphatidylinositol signaling system.

The expression of 6-phosphofructo-2-kinase (PFKFB1) was upregulated in the 15% fat diet with 1% taurine in comparison with the 15% fat diet, and the protein was enriched in the AMPK signaling pathway (Figure 5) in our current study. PFKFB1 is a bifunctional enzyme that catalyzes the synthesis and degradation of fructose 2,6-bisphosphate, acting as an allosteric activator of phosphofructokinase-1 and an inhibitor of fructose 1,6-bisphosphatase, which confers to PFKFB1 a key role in the control of glycolysis and gluconeogenesis [63]. Earlier studies have shown that upregulation of PFKFB1 expression is induced by AMPK activation [64]. AMPK stimulates catabolism by activating glucose uptake, glycolysis (due to PFKFB1 activation), glucose oxidation, and fatty acid oxidation [65]. Thus, the upregulation of PFKFB1 expression in the liver could be triggered by activated AMPK due to dietary taurine addition in the present study. The acetyl CoA carboxylase (ACC) and fatty acid synthase (FAS) are the key enzymes in fat synthesis [1], and were inhibited by activated AMPK in the liver of yellow catfish [66]. Therefore, the fat-reducing effect of dietary taurine in the liver of groupers could be achieved through degradation and utilization of glucose and fat by upregulating PFKFB1 expression through activating the AMPK signaling pathway in the present study.

To validate the reliability of RNA−Seq data, eight randomly selected DEGs in the taurine and high-fat comparison groups were examined using qRT−PCR. The fold changes obtained with qRT−PCR were consistent with the values obtained with RNA−seq for the six selected genes (*arf1_2*, *gk*, *atp1a*, *camk*, *cdo1*, and *cact*), suggesting our RNA-Seq data and the results based on RNA−Seq data analysis were reliable [22]. With association analysis of transcriptomic and proteomic data through KEGG, three DEGs and four DEPs were co-enriched into primary BA synthesis, bile secretion, the glycerolipid metabolism pathway, the phospholipid D signaling pathway, or/and the phosphatidylinositol signaling system. Compared with the 15% fat diet, taurine intervention upregulated the expression of the key protein CYP27α1 and the key gene *ATP1α*. Moreover, CYP27α1 and *ATP1α* were enriched into the primary BA synthesis and bile secretion pathways, respectively. The findings indicated that dietary taurine intervention helps promote BA synthesis in the liver and accelerates BA secretion and transport from the liver. It is well known that BAs have a good ability to assist in the digestion of fat in the intestine [52,54,58].

In the meantime, taurine intervention inhibited the expression of the key gene *arfi_2* and the key proteins LPIN, PLCD, and PTK2B in the liver of high-fat fed orange-spotted groupers which were co-enriched into the glycerol fat metabolism pathway, the phospholipase D signaling pathway, or/and the phosphatidylinositol signaling system pathway. Although the associated pathways varied, the metabolites of these pathways directly or indirectly point to DAG, which is known to be a precursor substance for TG synthesis [49]. In this sense, the reduction in TG accumulation in the liver was closely associated with the downregulation of key gene and key proteins caused by taurine intervention in high-fat fed groupers. In addition, we previously reported that the reduction in liver fat deposition caused by dietary taurine addition in high-fat fed groupers was the result of the decrease in the content of TG molecules at the lipidomic level [20]. The increased TG molecules may be achieved by simultaneously upregulating the expression of the key gene *arfi_2* and the expression of the key proteins LPIN and PLCD after taurine intervention. It is puzzling that we observed that the 15% fat diet with 1% taurine upregulated the expression of the key gene *plcd* and downregulated the expression of the key protein PLCD compared to the 15% fat diet in the present study, with inconsistent expression results of the same gene and protein. It is precisely because of the complexity of biological metabolic regulation that it is necessary for us to conduct in-depth analysis of the reasons for the inconsistent results in the future.

## 5. Conclusions

In the present study, new DEPs were identified and were enriched into metabolic pathways related to liver fat metabolism through proteomic analysis and its association with transcriptomic analysis. Three key DEGs (*atp1_a*, *arf1_2*, and *plcd*) and four key DEPs (CYP27α1, LPIN, PLCD, and PTK2B) were identified and co-enriched into the related pathways in liver fat metabolism. The expression changes in these key DEGs and DEPs were associated with increased BA biosynthesis and reduced TG biosynthesis, thereby mediating the effect of reducing fat in the liver after taurine intervention in high-fat fed orange-spotted groupers. To our knowledge, this study presents the first proteomic analysis and its integration analysis with transcriptomics on the fat-reducing effect of dietary taurine in the liver and reveals the close relationship between taurine and liver fat metabolism in the fish species. Further study will be carried out to investigate the functions and roles of these key proteins and key genes as target proteins and genes in the regulation of liver fat metabolism in fish in the future.

## Figures and Tables

**Table 1:** Ingredients and proximate composition of experimental diets (on an as−fed basis, %).

Ingredients	Diets (Fat Level/Taurine Level)		
	10F (10/0)	15F (15/0)	15FT (15/1)
Casein:gelatin = 4:1	50	50	50
Shrimp meal	4	4	4
Corn starch	25	25	25
Oil blend (fish oil:soy oil = 1:1)	6	10	10
Soy lecithin	4	4	4
Premix	0.8	0.8	0.8
Ca(H_2_PO_4_)_2_	2	2	2
Microcrystalline cellulose	7.2	3.2	2.2
Sodium alginate	1	1	1
Taurine	0	0	1
Nutrient level (analyzed values)			
Dry matter	91.18	90.24	90.38
Crude protein	46.55	46.87	46.56
Crude lipid	10.44	14.79	14.89
Taurine	0.04	0.04	0.98

**Table 2:** Protein expression profile in the TMT detection mode.

Protein ID	Protein Abbreviation	Protein Description	FC	TMT Pattern	PRM Pattern
TR5402_c1_g1_ORF	CYP27α1	Cholesterol 27-hydroxylase	1.564	up	up
TR511_c4_g1_ORF	PFKFB1	6-phosphofructo-2-kinase	1.269	up	up
TR314_c8_g1_ORF	PLCD	Phosphatidylinositol phospholipase C	0.792	down	down
TR944_c0_g1_ORF_1	ABAT	4-aminobutyrate aminotransferase	1.393	up	up
TR1786_c0_g1_ORF	GNAL	Guanine nucleotide-binding protein G subunit alpha	0.803	down	down
TR1499_c4_g2_ORF	GANC	Neutral alpha-glucosidase C	0.804	down	**nd**
TR2721_c0_g1_ORF	ENPP1_3	Ectonucleotide pyrophosphatase	0.785	down	down
TR244239_c0_g1_ORF	PCBD	4a-hydroxytetrahydrobiopterin dehydratase	1.319	up	up

**Table 3:** Identification result of differentially expressed proteins related to fat metabolism in 15% fat diet with 1% taurine group versus 15% fat diet group.

Protein ID	Map Name	Protein Description	FC	*p*-Value	Expression Pattern
TR5402_c1_g1_ORF	Primary bile acid biosynthesis	Cholesterol 27-hydroxylase	1.564	0.0181	Up
TR854_c2_g1_ORF_1	Glycerolipid metabolism/Glycerophospholipid metabolism/mTOR signaling pathway	Phosphatidate phosphatase LPIN	0.812	0.0114	Down
TR147_c2_g1_ORF	Glycerophospholipid metabolism/MAPK signaling pathway-yeast	Glycerol-3-phosphate dehydrogenase	0.726	0.0307	Down
TR2191_c0_g1_ORF	Glycerophospholipid metabolism	Phosphatidylserine sn-1 acylhydrolase	0.816	0.0393	Down
TR969_c0_g1_ORF_1	Sphingolipid metabolism	Ceramide synthetase	0.81	0.0217	Down
TR3417_c0_g1_ORF	Sphingolipid signaling pathway	transcription factor	0.822	0.00281	Down
TR1786_c0_g1_ORF	Calcium signaling pathway	Guanine nucleotide-binding protein G(olf) subunit alpha	0.803	0.0297	Down
TR1499_c4_g2_ORF	Galactose metabolism	Neutral alpha-glucosidase C	0.804	0.0303	Down
TR61997_c0_g2_ORF	Sphingolipid signaling pathway/FoxO signaling pathway	Mitogen-activated protein kinase	0.796	0.0119	Down
TR43919_c0_g1_ORF	Phospholipase D signaling pathway/Calcium signaling pathway	Focal adhesion kinase 2	0.797	0.0053	Down
TR52940_c0_g1_ORF	mTOR signaling pathway	Calcium binding protein	0.767	0.0443	Down
TR511_c4_g1_ORF	AMPK signaling pathway	6-phosphofructo-2-kinase	1.269	0.0153	Up
TR2721_c0_g1_ORF	Starch and sucrose metabolism	Ectonucleotide pyrophosphatase	0.785	0.0359	Down
TR244239_c0_g1_ORF	Folate biosynthesis	4a-hydroxytetrahydrobiopterin dehydratase	1.319	0.0351	Up
TR944_c0_g1_ORF_1	beta-Alanine metabolism	4-aminobutyrate aminotransferase	1.393	0.0101	Up
TR76930_c0_g1_ORF	Citrate cycle	Malate dehydrogenase	0.801	0.0486	Down
TR65955_c0_g1_ORF	Endocytosis	Arf-GAP with SH3 domain	0.825	0.0437	Down
TR314_c8_g1_ORF	Phosphatidylinositol signaling system/Calcium signaling pathway	Phosphatidylinositol phospholipase C	0.792	0.0403	Down

**Table 4:** KEGG pathways of the differentially expressed genes (DEGs) and proteins (DEPs) related in liver fat metabolism.

Map ID	Map Name	DEGs/DEPs
ko00120	Primary bile acid biosynthesis	CYP27α1
ko04976	Bile secretion	*ATP1α*
ko00561	Glycerolipid metabolism	LPIN
ko04072	Phospholipase D signaling pathway	*arf1_2*/PTK2B
ko04070	Phosphatidylinositol signaling system	*plcd*/PLCD

DEGs are represented in italics, while DEPs are represented in regular font.

## Data Availability

The data that support the findings of this study are available from the corresponding author upon reasonable request.
